# Effects of Exogenous Lactase Administration on Hydrogen Breath Excretion and Intestinal Symptoms in Patients Presenting Lactose Malabsorption and Intolerance

**DOI:** 10.1155/2014/680196

**Published:** 2014-05-25

**Authors:** Ivan Ibba, Agnese Gilli, Maria Francesca Boi, Paolo Usai

**Affiliations:** Department of Internal Medicine, Cagliari University, A.O.U. Policlinico di Monserrato, SS 554, 4,500 Km, 09042 Monserrato, Italy

## Abstract

*Objective.* To establish whether supplementation with a standard oral dose of Beta-Galactosidase affects hydrogen breath excretion in patients presenting with lactose malabsorption. *Methods.* Ninety-six consecutive patients positive to H2 Lactose Breath Test were enrolled. Mean peak H2 levels, the time to reach the peak H2, the time to reach the cut-off value of 20 ppm, the cumulative breath H2 excretion, the areas under the curve, and a Visual Analogical 10-point Scale for symptoms were calculated. Genotyping of the C/T-13910 variant was carried out. *Results.* Following the oral administration of Beta-Galactosidase, in 21.88% of the cases, H2 Lactose Breath Test became negative (Group A), while mean peak H2 levels (74.95 ppm versus 7.85), *P* < 0.0000, in 17.71% (Group B) were still positive, with the H2 level 20 ppm above the baseline, but the peak H2 levels were significantly lower than those observed at the baseline test (186.7 ppm versus 66.64), *P* < 0.0000, while in 60.41% (Group C) they were still positive with the peak H2 levels similar to those observed at the baseline test (94.43 versus 81.60 ppm). All 96 individuals tested presented the C/C-13910 genotype nonpersistence. *Conclusions.* The response to oral administration of Beta-Galactosidase in patients with symptoms of lactose malabsorption presents a significant variability.

## 1. Introduction

Lactose malabsorption (LM), intolerance (LI) are a common condition affecting a large proportion of the world's population [1]. The most common cause of lactose intolerance is lactase deficiency, a decreased production of the enzyme lactase in the small intestinal villi. In normal conditions, lactose is broken down in the small intestine, by lactase, to glucose and galactose [2]. Lactase-deficient individuals are not able to cleave this disaccharide and may become symptomatic following the ingestion of lactose. In LM, undigested lactose is fermented by the colonic flora causing, in many subjects, symptoms of LI such as diarrhoea, bloating, nausea, borborygmi, and abdominal pain.

Several treatment approaches have been proposed over the last few years [3], namely, addition of exogenous lactase to intact milk [4, 5], low-lactose milk [6], yogurt, and probiotics, due to their bacterial lactase activity [7–9] and pharmacological and nonpharmacological strategies that can prolong the contact time between enzyme and substrate delaying gastrointestinal (GI) transit time [10] and lactose administration for colonic adaptation [11]. Enzyme-replacement treatment with microbial exogenous lactase (obtained from yeasts or fungi) represents a possible strategy for primary lactase deficiency. However, while several studies have confirmed the efficacy of solid lactase preparations in reducing both H2 excretion and symptoms [12, 13], other comparative studies have shown that these preparations are significantly less effective than prehydrolysed milk, probably due to the gastric enzyme inactivation [14]. Since LI management with Beta-Galactosidase oral supplements remains unclear and data regarding their efficacy in reducing the H2 breath concentration are inadequate, the aim of the present investigation was to assess whether supplementation with a standard oral dose of Beta-Galactosidase obtained from* Aspergillus oryzae* affects hydrogen breath excretion and GI symptoms in lactose intolerant patients.

## 2. Materials and Methods

### 2.1. Patients

After approval of our Ethical Committee, we have selected, between January 2011 and June 2011, 96 consecutive patients (80 females and 16 males, overall mean age 38.0 years, range 18–65) who attended to the Gastroenterology Outpatient Unit of University of Cagliari, Italy, for the presence of GI symptoms, abdominal pain, nausea, bloating, and borborygmi, following lactose ingestion and were evaluated for LM by means of H2 Lactose Breath Test (H2 LBT). All patients were positive to H2 Lactose Breath Test and were considered eligible for the study following a detailed explanation regarding the investigation. All patients agreeing to take part, following a detailed explanation regarding the investigation, signed an informed consent form before admission. Exclusion criteria were age <18 or >65 years, diagnosis of neoplasia, inflammatory bowel disease, previous GI surgery, history of allergy to milk proteins, lack of compliance, history of liver, kidney, lung, heart, metabolic, or neurological disorders, treatment with laxatives, antibiotics, and prokinetics, or any other treatment known to affect the colonic flora or motility in the month prior to the study. Patients were interviewed regarding GI symptoms (abdominal pain, nausea, bloating, and diarrhoea) and completed a questionnaire, including items regarding demographic data [e.g., sex, age, and body mass index (BMI)].

### 2.2. H2 Lactose Breath Test

The H2 LBT was performed using a breath gas analyzer Model 12i MicroLyzer Plus (Quintron Instruments, Milwaukee, WI, USA). Basal breath specimens were obtained after overnight fasting; the day before each breath test, the patients avoided eating slowly absorbed carbohydrates (bread, pasta, or fibre) in order to avoid delayed exhalation of hydrogen in the breath [15]. Cigarette and/or cigar smoking and physical exercise were not permitted within the 12 hours before the test in order to prevent hyperventilation and consequent changes in hydrogen content in the breath. Before starting the test, patients rinsed their mouths with an antiseptic wash (Chlorhexidine 0.05% 20 mL), followed by tap water, to avoid a rapid hydrogen peak due to the effect of oral bacteria on lactose. After having evaluated the baseline H2 breath concentration, the patients swallowed 25 gr of lactose (equivalent of the lactose content in 500 mL of cow's milk) dissolved in 300 mL of water. Over a 4-hour period, breath samples were collected at 30-minute (min) intervals (from 8 a.m. till 12 noon) by having the subjects exhale into a mylar foil gas two-bag system while the patient was in a sitting position [16, 17]. The H2 LBT, in agreement with the last International Guidelines (see, e.g., Rome Consensus Conference, 2007), was considered positive for lactose malabsorption if the H2 concentration, in the exhaled air, exceeded 20 parts per million (ppm) above the baseline during the monitoring period [18, 19]. Mean peak H2 levels (ppm), the time to peak H2 (min), and the time to reach the cut-off value of 20 ppm were calculated [20, 21]. Also, to better standardize data collection and further strengthen the hypothesis of our study, we evaluated the cumulative breath H2 excretion (ppm). We also assessed the value of the areas under the entire curve (overall concentration of exhaled H2 in 4 hours). Two H2 LBTs were carried out in each patient, the initial test and the test following intervention (15000 Units of an acid-resistant Beta-Galactosidase obtained from* Aspergillus oryzae*), and were administered 1 hour before lactose intake. As pointed out by the manufacturer, one tablet (7500 Units) is able to hydrolyse 16 gr of lactose; thus, 2 tablets (equal to 15000 Units) should be sufficient to hydrolyse 25 gr of lactose contained in the solution administered to the patient. To avoid the effect of colonic acidification, the mean time interval between the baseline test and following the intervention was 8 days (range 9-10 days) [22].

### 2.3. Gastrointestinal Symptoms

On the day of the test, during the 8 hours after substrate ingestion, all patients were asked to rate four symptoms (abdominal pain, nausea, bloating, and borborygmi) using a Visual Analogical 10-point Scale (VAS) (0, no symptoms, to 10, the severity of the symptom). For each patient, the VAS was calculated for each symptom as well as the cumulative VAS by adding together the single symptom VAS score of levels.

### 2.4. Genotyping

DNA was isolated from EDTA-blood using a QiaAmp blood DNA Extraction kit (Qiagen, Hilden, Germany). Briefly, 200 lL EDTA-blood was treated with protease for 15 min at 56°C followed by addition of AL lysis buffer and ethanol. The mixture was passed through a spin column and washed according to the manufacturer's instructions. DNA was eluted with 100 l-LAE buffer and quantified on agarose gel using lambda DNA as the standard. The DNA fragment spanning C/T-13910 variants were amplified using the forward primer [5#-GGA TGC ACT GCT GTG ATG AG-3#] and reverse primer [5#-CCC ACT GAC CTA TCC TCG TG-3#] to also include positions.

Both sequencing and RFLP were carried out by using this PCR product, which was done without knowledge of the clinical data and the results of the LTT and LBT.

### 2.5. Sequencing

The PCR product was sequenced using an automated DNA Sequencer (ABI3100 Applied Biosystems, Ipswich, MA, USA) with the forward primer to read 400 base pairs (bps) in one direction. When necessary, the result was reconfirmed by sequencing the other strand with the reverse primer. All sequence data could be read with a high confidence level from 213830 to 214190 bps (i.e., 361 bps spanning the C/T13910 upstream of the LCT locus). MCM6 reference gene sequence (GenBank reference sequence) was used because this SNP lies within the MCM6 locus, which is the neighbouring from the gene upstream of the LCT locus.

### 2.6. Restriction Fragment Length Polymorphism

The PCR product (300 ng) was digested with 1-2 Units of BsmFI restriction enzyme (New England Biolabs, Foster City, CA, USA) and 1*·*reaction buffer B in a 30 lL reaction volume. The reaction mixture was incubated at 65°C for 4 hours and then electrophoresed on a 2.5% agarose gel, stained with ethidium bromide, and visualized under ultraviolet light (302 nm). On the basis of the sequence information around the 13910 upstream position of the LCT locus, the expected band pattern was as follows: C/C variant: 342 and 106 bps (2 bands); C/T variant: 342, 224, 118, and 106 bps (seen as 3 bands on the gel as the 106 bps and 118 bps were not usually well resolved); and the T/T variant: 224, 118, and 106 bps (seen as 2 bands, for the same reason); the size of the uncut PCR product was 448 bps (one band).

### 2.7. Statistical Analysis

The statistics data were analysed using SPSS software. We performed a test of normality on the quantitative variables mean peak H2 levels (ppm), the time to peak H2 (min), the time to reach the cut-off value of 20 ppm, and the cumulative breath H2 excretion (ppm) to define the type of test for the hypothesis testing. Tests of normality were significant; therefore, we proceeded with nonparametric tests for testing hypotheses about the variables mean peak H2 levels (ppm), the time to peak H2 (min), the time to reach the cut-off value of 20 ppm, the cumulative breath H2 excretion (ppm), and the areas under the curve. Scores of abdominal pain, nausea, bloating and diarrhoea compared before-after and were tested with the Kruskal-Wallis test. To avoid spurious assessment of statistical significance between groups differences, we proceeded to analyze the data with ANOVA. In particular, we tested simultaneously Groups A, B, and C before and after oral intake of Beta-Galactosidase for the mean peak H2 levels (ppm), the time to peak H2 (min), the time to reach the cut-off value of 20 ppm, and the cumulative breath H2 excretion (ppm).

## 3. Results

**Figure 1:**
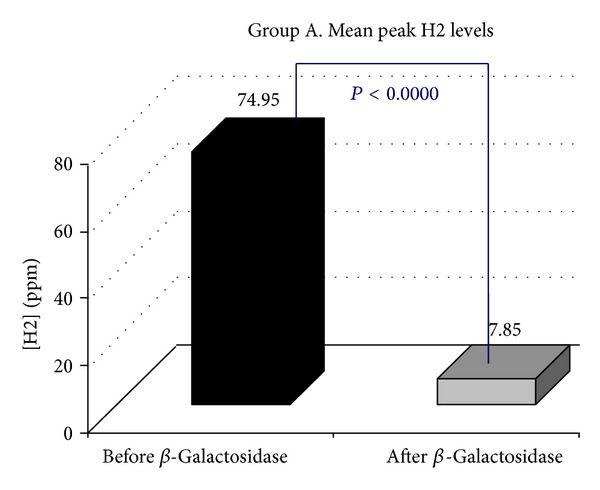


**Figure 2:**
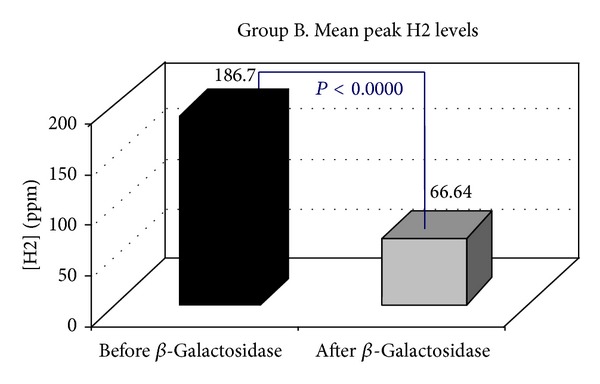


**Figure 3:**
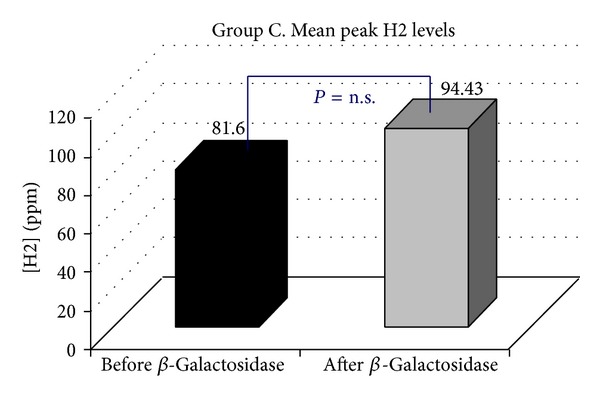


**Figure 4:**
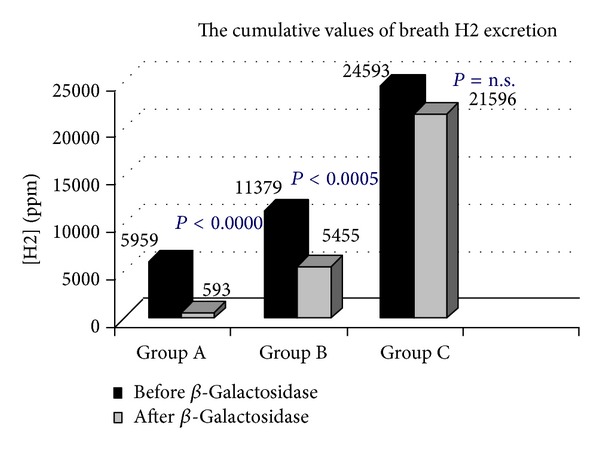


**Figure 5:**
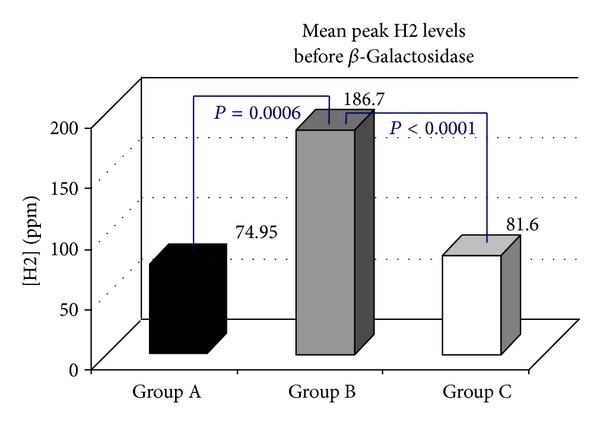


**Figure 6:**
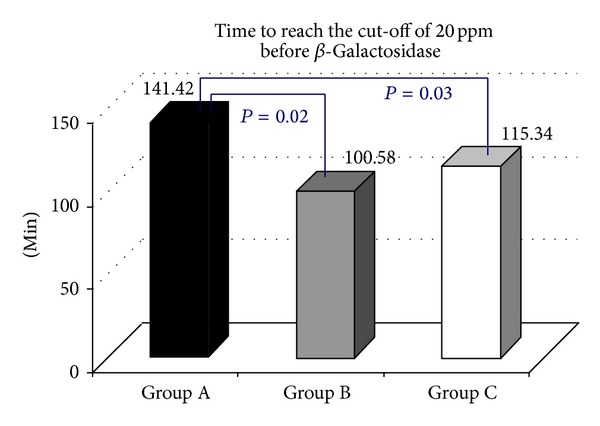


**Figure 7:**
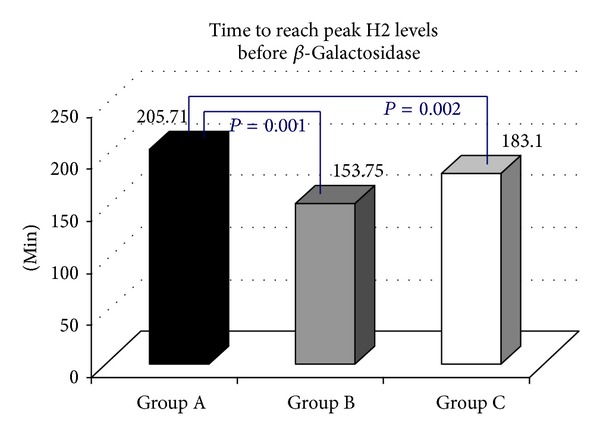


Following the oral administration of tilactase, in 21/96 (21.88%) H2 LBT became negative (Group A, Figure 1), while mean peak H2 levels (74.95 versus 7.85 ppm) *P* < 0.0000, in 17/96 (17.71% Group B, Figure 2) were still positive with H2 levels 20 ppm above the baseline but the mean peak H2 levels were significantly lower than those observed at the baseline test (186.7 versus 66.64 ppm) *P* < 0.0000, while 58/96 (60.41% Group C, Figure 3) were still positive with mean peak H2 levels similar to those observed at the baseline test (81.60 versus 94.43 ppm). We also observed that the cumulative values of breath H2 excretion (Figure 4) were significantly reduced after Beta-Galactosidase administration in Group A (5959 versus 593 ppm), *P* < 0.0000, and in Group B (11379 versus 5455), *P* < 0.0005. There was no significant reduction in Group C (24593 versus 21596 ppm) after Beta-Galactosidase administration. At the baseline H2 LBT (after ingestion only of lactose), in Group A, the mean peak H2 levels (Figure 5) were significantly lower than in B (74.95 versus 186.7 ppm), *P* = 0.0006; furthermore, the time to reach the cut-off value of 20 ppm (Figure 6) was significantly longer in Group A than that observed in Group B (141.42 versus 100.58 min), *P* = 0.02, and Group C (141.42 versus 115.34 min), *P* = 0.03, and the time to reach the peak H2 levels (Figure 7) was significantly longer in A than in B (205.71 versus 153.75 min), *P* = 0.001, and Group C (205.71 versus 183.10 min), *P* = 0.002. No statistically significant result was achieved by the analysis of the areas under the entire curve before and after Beta-Galactosidase administration.

All 96 individuals tested presented the C/C-13910 genotype, which is the polymorphism for the Sardinian population associated with lactase nonpersistence [23].

### 3.1. Effect of Tilactase on Symptoms

Following the oral tilactase consumption, a significant reduction in the mean clinical scores for abdominal pain, bloating, and diarrhoea was observed in Group C; in Group B, a significant reduction in the mean clinical score resulted in nausea and diarrhoea, while in Group A a significant reduction in the mean clinical score resulted in bloating (Table 1).

## 4. Discussion

Results of the present study indicate that oral administration of 15000 Units (600 Units × gr lactose) of Beta-Galactosidase obtained from fermentation of* Aspergillus oryzae*, followed by ingestion of a water lactose solution, in lactose malabsorber individuals, with genetically related hypolactasia with the C/T-13910 variant and GI symptoms of LI, is effective in significantly decreasing the mean peak H2 levels and the cumulative values of breath H2 excretion in approximately 40% of subjects (Groups A and B), while, in 60% of subjects (Group C), the breath H2 excretion levels do not change with respect to the baseline test. The value of the areas under the entire curve could play a predictive role regarding the test response after administration of lactase in order to understand how a greater or lesser concentration of global H2 breath could affect the outcome of the test in relation to the amount of lactase administered. However, no significant result was observed after statistical analysis. Regarding the effect of tilactase ingestion on GI symptoms, a significant reduction in the symptom score with the exception of nausea was observed. It is noteworthy that these data indicate a significant variability in the responses to oral Beta-Galactosidase. Our study, in agreement with other works, has shown that there is no direct correlation between symptoms and H2 breath excretion. We also observed that the clinical response after administration of a standard dose of lactase does not correlate directly with the H2 concentration in exhaled air. This fact is particularly evident in Group C in which, after taking lactase, any substantial change is not observed with regard to the mean peak H2 levels and the cumulative breath H2 excretion values. Also, though interesting, unfortunately, our work did not take into account body weight and BMI value of patients; in fact, many studies have been conducted with the aim of evaluating the relationship between body weight and the dose required for a positive effect of tilactase. A possible explanation for the interindividual differences could be the effect of variations in the degree of lactose digestion (LD) [24], of the potential gastric inactivation of the enzyme [25], of the intestinal motility patterns [26], or of the gastric emptying [27]. At the baseline test, some significant differences between the groups were present; for instance, in Group A, we detected a longer time to reach the cut-off value of 20 ppm and the time to reach the peak H2 levels. In Group A, a delay in the orocecal transit time could possibly be a possible explanation for the longer time to reach the cut-off value as well as the time to reach the peak H2 levels. A delay in the small intestine transit time suggests that longer exposure between the Beta-Galactosidase and the lactose in the intestinal lumen could contribute to improve the LD [28–31]. Furthermore, the decrease in intestinal transit time, prolonging the action of the Beta-Galactosidase in the intestinal lumen, decreases, in turn, the osmotic load of the lactose which, as a nonabsorbable sugar, could accelerate the intestinal transit time reducing the time available for lactose hydrolysis [30–32]. However, the transit time is not the only explanation behind the different responses obtained in the three groups after taking lactase. As discussed, other mechanisms, not better known and currently unconvincing, could play a role.

In Group B, following Beta-Galactosidase, the H2 LBT was still positive, with H2 levels 20 ppm above baseline, and the peak H2 levels and the cumulative breath H2 excretion were, however, significantly lower than those observed at the baseline H2 LBT. Of note in this group is the fact that, at baseline H2 LBT, the mean peak levels of H2 were significantly higher than those at Groups A and C. In this group, the significant but partial response to Beta-Galactosidase could be due to a lower concentration of the epithelial enzyme, and therefore higher levels of exhaled H2 could be achieved by the larger amount of lactose reaching the colonic lumen where it is fermented by the flora in the colon. In these hypotheses, a higher dose of oral Beta-Galactosidase could be a useful tool for increasing the hydrolysis of ingested lactose in the small bowel, thus reducing the amount of undigested lactose reaching the large intestine. However, the high levels of exhaled H2, observed in Group B, could also be the result of the colonic bacteria Beta-Galactosidase activity or the amount of methanogenic bacteria present in the colon. The hydrogen produced following lactose ingestion, by lactose-intolerant patients, is likely, at different rates, oxidised by methanogenic bacteria; therefore, it could be argued that, in Group B, nonsignificant amounts of H2 are consumed by methanogenic and/or sulphate-reducing bacteria. Therefore, an interindividual variability in the microbiota and in the colonic bacteria Beta-Galactosidase activity is possibly involved in determining the difference in the amount of H2 in the lactose colonic fermentation. For these reasons, in some cases, an oral supplementation of oral lactase (over 15000 Units) could reduce the concentration of H2 expired in no responder patients (Group C) or modify the severity of symptoms in Groups A and C. In approximately 60% of patients (Group C), the oral administration of Beta-Galactosidase was not effective in decreasing breath H2 excretion. The “resistance” to the oral Beta-Galactosidase observed in these patients could be the result of inactivation of the exogenous enzyme. The Beta-Galactosidase, in order to effectively maintain the enzymatic activity in the conditions usually found in the gastrointestinal tract such as gastric acidity and bile concentrations, requires the mechanical protection of the enzyme during the gastric passage and against the action of the bile [33]. It has been demonstrated that gastric acid reduces bacterial lactase activity in 20–60 min [34]. If the mechanical protection of the enzyme is disgregated in the gastric lumen, the acid pH could reduce the action of the residual Beta-Galactosidase. Gastric emptying and intestinal transit should also be taken into consideration; a fast gastric emptying and intestinal transit time with consequently a shorter contact time between enzyme and substrate could reduce the carbohydrate absorption. Studies that evaluated the effect of propantheline and metoclopramide on lactose digestion revealed that propantheline-induced prolongation of gastric emptying improves lactose tolerance as measured by breath H2 concentration compared to metoclopramide [35]. It has already been hypothesized that the levels of breath H2 excretion might influence the occurrence of symptoms following lactose ingestion [36, 37].

In this respect, in the present series, the decrease in H2 levels in the breath does not seem to affect the symptom scores; in fact, no significant positive correlation was found between the peak H2 levels and the total symptom score, in particular for abdominal pain and nausea.

The present data are in agreement with those by di Stefano et al. [38] who found no correlation between the severity of symptoms and the level of breath H2 excretion. The causes of the GI symptoms in lactose intolerant patients are not clearly understood. Several factors could contribute to the development of symptoms, for instance, psychological factors [39], functional GI disorders, visceral sensitivity, or bowel motor abnormalities [40]. This would appear to suggest that, in addition to the digestion of lactose in the small intestine, other factors may influence the onset of lactose intolerance symptoms. Recently, the involvement of colonic factors has been hypothesized [41–44]. The balance between the ability of the colonic microbiota to ferment lactose and the ability of the colon to remove the fermentation metabolites would influence the onset of lactose intolerance, making it either more severe or less severe. A low lactose fermenting capacity of the colonic microbiota, which leads to inefficient removal of maldigested lactose (and/or its intermediate fermentation metabolites, e.g., glucose and galactose) or to a low absorption capacity of the colon or a low SCFA/gas-metabolizing capacity of the colonic microbiota which leads to poor removal of fermentation metabolites, may contribute to the development of symptoms. Although, following oral Beta-Galactosidase administration, an improvement in some GI symptoms, bloating in Group A and nausea and diarrhoea in Group B, was obtained, our findings show that, particularly in patients not presenting a decrease in the H2 levels following oral Beta-Galactosidase administration (Group C), a more extensive improvement in abdominal symptoms (abdominal pain, bloating, and diarrhoea) was observed. At the moment, we have no security if the improvement in the severity of symptoms observed in Group C is the result of a placebo effect or different metabolic response to lactase. Our results indicate that oral administration of 15000 Units of Beta-Galactosidase in lactose malabsorber individuals is effective in decreasing significantly the mean peak H2 levels and the cumulative values of breath H2 excretion in a small group of patients. However, this reduction may not be directly correlated with the severity of symptoms. It is plausible, therefore, that other factors play an important role in the proper metabolism of tilactase and the real benefits of the same after oral intake. Further studies are needed to elicit the different impact of oral administration of Beta-Galactosidase on GI symptoms and on breath excretion of H2.

## Figures and Tables

**Table 1:** Visual analogical scale for symptoms.

	Abdominal pain	Bloating	Nausea	Diarrhoea
Group A				
Before Beta-Galactosidase	2.54	5.02	1.62	0.88
After Beta-Galactosidase	2.07	3.42*	1.09	0.28
		*P = 0.02		
Group B				
Before Beta-Galactosidase	3.61	5.29	0.73	1.53
After Beta-Galactosidase	3.50	5.11	0.14*	0.53*
			*P = 0.04	*P = 0.02
Group C				
Before Beta-Galactosidase	3.68	5.35	1.55	1.74
After Beta-Galactosidase	2.78*	4.47*	1.13	1.00*
	*P = 0.01	*P = 0.02		*P = 0.003
